# Comparison of new metal organic framework-based catalysts for oxygen reduction reaction

**DOI:** 10.1016/j.dib.2018.05.011

**Published:** 2018-05-08

**Authors:** Shmuel Gonen, Lior Elbaz

**Affiliations:** Institute of Nanotechnology and Advanced Materials, Department of Chemistry, Bar-Ilan university, 5290002 Ramat Gan, Israel

## Abstract

In this article, we collected the most significant and recent data in brief in the field of metal organic frameworks oxygen reduction reaction catalysts, obtained from some of the most recent research papers in the field. We present lists of materials and their key parameters that are relevant to the cathode catalysts in polymer electrolyte membrane fuel cells. All the materials listed in this paper are composed of metal organic frameworks, zeolitic imidazolate frameworks, or their derivatives. These are divided into two main groups: pristine MOFs and MOF-derived materials. The data in this article is a summary of more extensive review (Gonen and Elbaz, 2018) [1].

## Specifications Table

**Table:** 

Subject area	*Electrochemistry*
More specific subject area	*Electrocatalysis; Oxygen Reduction; Fuel Cells*
Type of data	Table 1, Table 2
How data was acquired	*Survey of current literature*
Data format	*Summary*
Experimental factors	*Heat treatment temperature, pH, Onset potential, Half-wave potential, peak power*
Experimental features	*Reported values*
Data source location	*Cited articles*
Data accessibility	*The data is located in several scientific papers*[1]*. Full details of the sources can be found in the bibliography.*

## Value of the data

• The data in here is extensive, and summarizes the activity of some of the most active metal organic frameworks (MOF) catalysts for oxygen reduction reaction (ORR).
• It contains the most important catalytic parameters, as well as the conditions and treatments, therefore can be served as a benchmark for comparison of any new MOFs or other platinum group-free (PGM-free) ORR catalyst
• The tables distinguish between the two main types of catalysts in this field, pristine MOFs and MOF-derived catalysts (thermally treated), in order to avoid confusion.
• From the data, researchers can extract influences and trends in fuel cells catalysis, and conclude which materials have the best potential for their study and applications.

## 1 Data

See Table 1, Table 2.

**Table 1:** MOF ORR catalysts (supported or pristine).

**Acronym**	**Name**	**Support**	**Testing pH**	***E***_***onset***_	***E***_***1/2***_	***P***_***max***_	**Refs.**
**CuS@Cu-BTC**	CuS@Cu-BTC	CuS	0.1 M KOH (13)	0.91 V vs. RHE	–	–	[2]
**MOF(Fe)**	Fe-BTC	SP carbon	0.1 M KOH (13)	− 0.12 V vs. Ag/AgCl	–	–	[3]
**MOF(Fe/Co)**	(Fe/Co)-BTC	SP carbon	0.1 M KOH (13)	− 0.13 V vs. Ag/AgCl	–	–	[4]
**Cu-BDC-TED**	Cu-(BDC + triethylene-diamine) GO	Graphene Oxide	0.5 M H_2_SO_4_ (0)	0.29 V vs. RHE	–	110.5 mW cm^−2^	[5]
**NPC-4**	Cu_2_(TMBDI)(H_2_O)_2_	rGO	0.1 M phosphate buffer (6)	− 0.13 V vs. Ag/AgCl	–	–	[6]
**Ni-CAT**	Ni-catecholate framework	SP carbon	0.1 M KClO_4_ and 0.02 M PBS (7)	–	− 0.236 V vs. Ag/AgCl	–	[7]
**Ni-CAT**	Ni-catecholate framework	SP carbon	0.1 M KOH (13)	–	− 0.196 V vs. Ag/AgCl	–	[7]
**[Co(bpy)**_**3**_**](N O**_**3**_**)**_**2**_	[Co(bipyridine)_3_](NO_3_)_2_	Ketjenblack	0.1 M KOH (13)	0.8 V vs. RHE	–	–	[8]
**Co-OBA**	Co-Oxybis (benzoic acid)	Vulcan XC-72	0.1 M KOH (13)	− 0.197 V vs. Ag/AgCl	–	–	[9]
**Co/MIL-101(Cr)**	Co(Cr-BDC)	Vulcan XC-72	0.1 M KOH (13)	− 0.05 V vs. Ag/AgCl	− 0.33 V vs. Ag/AgCl		[10]
**Co-MOF**	Co-benzimidazolate	CNTs	0.1 M KOH (13)	0.91 V vs. RHE	0.82 V vs. RHE	–	[11]
**ZIF-67**	Co-methyl-imidazolate	pomelo-peel-derived carbon	0.1 M KOH (13)	–	0.82 V vs. RHE	–	[12]
**Cu(phen-NO**_**3**_**)(BTC)**	Cu(nitrophenanthroline)(BTC)	CNTs@TiO_2_	0.1 M KOH (13)	0.988 V vs. RHE	0.805 V vs. RHE	–	[13]
**PCN-223-Fe**	Zr_6_O_4_(OH)_4_(Fe(III)-(TCPP)_3_)	None	0.1 M LiClO_4_/DMF	− 0.5 V vs NHE	− 0.56 V vs NHE	–	[14]
**Ni**_**3**_**(HITP)**_**2**_	Ni_3_(hexaiminotriphenylene)_2_	None	0.1 M KOH (13)^a^	0.82 V vs. RHE	–	–	[15], [16]
**Pt 20%/XC-72**	Pt 20%/XC-72	Vulcan XC-72	0.5 M H_2_SO_4_ (0)	0.9 V vs. RHE	0.81 V vs. RHE	–	[17]

a Active at different pH values as well.

**Table 2:** MOF-derived ORR catalysts (heat treated).

**Acronym**	**Name**	**Heat treatment temperature (°C)**	**Testing pH**	***E***_***onset***_	***E***_***1/2***_	***P***_***max***_	**Ref.**
**Co-Im**	Co-Imidazolate	750	0.1 M HClO_4_ (1)	0.83 V vs. RHE	0.68 V vs. RHE	–	[18]
**Fe/Phen/Z8**	Fe-phenanthroline/ZIF-8	1050 (Ar), 950 (NH_3_)	acid	–	–	910 mW cm^−^^2^	[19]
**PB**	Prussian blue	800	0.1 M KOH (13)	0.95 V vs. RHE	0.82 V vs. RHE	–	[20]
**Co-MOF**	Co-BTC	900^a^	0.1 M KOH (13)	0.88 V vs. RHE	–	–	[21]
**Fe/IRMOF-3**	Fe(Zn-NH_2_-BDC)	900	0.1 M NaOH (13)	1.02 V vs. RHE	0.88 V vs. RHE	–	[22]
**MOF-253**	Fe-Al(OH)(bpydc)	900	0.1 M KOH (13) b	0.98 V vs. RHE	0.84 V vs. RHE	–	[23]
**Co-TA**	Co-polyphenol	800	0.1 M KOH (13)	0.98 V vs. RHE	–	–	[24]
**Fe-NH**_**2**_**-MIL-101**	Fe-NH_2_-MIL-101	700	0.1 M KOH (13)	0.99 V vs. RHE	0.84 V vs. RHE	–	[25]
**Fe-NH**_**2**_**-MIL-101**	Fe-NH_2_-MIL-101	700	0.5 M H_2_SO_4_ (0)	0.92 V vs. RHE	0.67 V vs. RHE	–	[25]
**Co**_**3**_**(PO**_**4**_**)**_**2**_**C-N/rGOA**	Co_3_(O_3_PCH_2_–NC_4_H_7_–CO_2_)_2_	800	0.1–1 M KOH (13–14)	0.968 V vs. RHE	0.872 V vs. RHE	–	[26]
**NiCoTU@NH**_**2**_**-MIL-101(Al)**	NiCo-thiourea-NH_2_-MIL-101(Al)	900	0.1 M KOH (13)	0.94 V vs. RHE	0.86 V vs. RHE	261.3 mW cm^−2^	[27]
**MIL-101-Fe**	Fe-aniline-BDC	900	0.1 M KOH (13)	0.058 V vs. Hg/HgO	–	–	[28]
**Fe-ZIF-8**	Fe-Zn-mIm		0.1 M HClO_4_ (1)	0.95 V vs. RHE	0.82 V vs. RHE	–	[29]
**Fe-ZIF-8**	Fe-Zn-mIm	1.1050 (Ar) 2. 1050 (NH_3_)	0.1 M HClO_4_ (1)	0.98 V vs. RHE	0.78 V vs. RHE	–	[30]
**Fe-ZIF-8**	Fe-Zn-mIm	1.1050 (Ar) 2. 1050 (NH_3_)	0.1 M KOH (13)	1.05 V vs. RHE	0.87 V vs. RHE	–	[30]
**Fe-ZIF-8**	Fe-Zn-mIm, Fe-Zn-Im	1.1050 (Ar) 2. 1050 (NH_3_)	0.1 M HClO_4_ (1)	0.91 V vs. RHE	0.778 V vs. RHE	668.8 mW cm^−2^	[31]
**CoCO-Pz**	Co-pyrazinedicarboxylate	700	0.5 M H_2_SO_4_ (0)^b^	0.97 V vs. RHE	0.72 V vs. RHE	60 mW cm^−2^	[32]
**ZIF-67**	Co-mIm	700	0.1 M KOH (13) b	0.97 V vs. RHE	0.87 V vs. RHE	–	[33]
**ZIF-67/ZIF-8**	Co-mIm/Zn-mIm	900	0.1 M KOH (13)	0.982 V vs. RHE	0.881 V vs. RHE	–	[34]
**ZIF-67/ZIF-8**	Co-mIm/Zn-mIm	850	0.1 M KOH (13)	0.992 V vs. RHE	0.91 V vs. RHE	–	[35]
**ZIF-67**	Co-mIm	1.800 (H_2_) 2. 250 (O_2_)	0.1 M KOH (13)	–	0.83 V vs. RHE	–	[36]
**ZIF-67**	Co-mIm	900	0.1 M KOH (13)	0.94 V vs. RHE	0.8 V vs. RHE	–	[37]
**S-ZIF-67**	Co-mIm-S	700	0.1 M KOH (13)	0.97 V vs. RHE	0.9 V vs. RHE	–	[38]
**S-ZIF-67**	Co-mIm-S	700	0.1 M HClO_4_ (1)	0.9 V vs. RHE	0.78 V vs. RHE	–	[39]
**S-ZIF-67**	Co-mIm-S	700	0.1 M KOH (13)	0.98 V vs. RHE	0.88 V vs. RHE	–	[39]
**ZIF-67**	Co-mIm	800	0.1 M KOH (13)	0.938 V vs. RHE	0.869 V vs. RHE	–	[40]
**ZIF-67/ZIF-8**	Co-mIm/Zn-mIm	950	0.1 M KOH (13)	1.0 V vs. RHE	0.87 V vs. RHE	–	[41]
**Fe-ZIF-8**	Fe-Zn-mIm	950	0.1 M KOH (13)^b^	0.975 V vs. RHE	0.867 V vs. RHE	–	[42]
**Fe-ZIF-8**	Fe-pyrrole-Zn-mIm	800	0.1 M KOH (13) b	0.96 V vs. RHE	0.83 V vs. RHE	–	[43]
**Fe-ZIF-8**	Fe-Zn-mIm	950	0.1 M HClO_4_ (1)	0.95 V vs. RHE	0.81 V vs. RHE	820 mW cm^−^^2^	[44]
**Fe-ZIF-8**	Fe-Zn-mIm	900	0.5 M H_2_SO_4_ (0)	0.861 V vs. RHE	0.735 V vs. RHE	–	[45]
**Fe-ZIF-8**	Fe-Zn-mIm	1.1050 (Ar) 2. 750 (NH_3_)	acid	–	–	603.3 mW cm^−2^	[46]
**Pt 20%/XC-72**	Pt 20%/XC-72	–	0.5 M H_2_SO_4_ (0)	0.9 V vs. RHE	0.81 V vs. RHE	–	[17]

a Different temperatures gave similar results.

b Was also measured in other electrolytes.

## 2 Experimental design, materials and methods

The onset and half wave potentials (**E**_**onset**_ and **E**_**1/2**_) were acquired by rotating disk electrode (RDE) measurements. RDE is conducted with three electrodes system when the studied material deposited on a disk working electrode with binder. The maximum power (**P**_**max**_) was acquired by single fuel cell measurement, in which a catalyst layer is deposited on a membrane to form a membrane electrode assembly (MEA). Maximum power is the peak power that is calculated from IV measurement.
