# Supplementary material for: Comparison of new metal organic framework-based catalysts for oxygen reduction reaction
Source: Data Brief. 2018 May 8;19:281–7. doi: 10.1016/j.dib.2018.05.011 (PMC5992994; doi:10.1016/j.dib.2018.05.011)
Supplement: Supplementary file 1 — Supplementary material [file mmc1.docx]

Conflict of Interest

The authors declare no conflicts of Interest.
